# Simulation of pressure support for spontaneous breathing trials in neonates

**DOI:** 10.1186/s40635-019-0223-8

**Published:** 2019-02-08

**Authors:** Makoto Sasaki, Yoshikazu Yamaguchi, Tetsuya Miyashita, Yuko Matsuda, Masahide Ohtsuka, Osamu Yamaguchi, Takahisa Goto

**Affiliations:** 1 Department of Anesthesiology and Critical Care Medicine, Yokohama City University, 3-9 Fukuura, Kanazawa-ku, Yokohama-shi, Kanagawa 236-004 Japan; 2 Department of Critical Care Medicine, Yokohama City University Medical Center, Yokohama, Japan

**Keywords:** Airway extubation, Mechanical ventilators, Neonate, Pulmonary ventilation, Ventilator weaning, Work of breathing

## Abstract

**Background:**

Endotracheal tubes used for neonates are not as resistant to breathing as originally anticipated; therefore, spontaneous breathing trials (SBTs) with continuous positive airway pressure (CPAP), without pressure support (PS), are recommended. However, PS extubation criteria have predetermined pressure values for each endotracheal tube diameter (PS 10 cmH_2_O with 3.0- and 3.5-mm tubes or PS 8 cmH_2_O with 4.0-mm tubes). This study aimed to assess the validity of these SBT criteria for neonates, using an artificial lung simulator, ASL 5000™ lung simulator, and a SERVO-i Universal™ ventilator (minute volume, 240–360 mL/kg/min; tidal volume, 30 mL; respiratory rate, 24–36/min; lung compliance, 0.5 mL/cmH_2_O/kg; resistance, 40 cmH_2_O/L/s) in an intensive care unit. We simulated a spontaneous breathing test in a 3-kg neonate after cardiac surgery with 3.0–3.5-mm endotracheal tubes. We measured the work of breathing (WOB), trigger work, and parameters of pressure support ventilation (PSV), T-piece breathing, or ASL 5000™ alone.

**Results:**

WOB displayed respiratory rate dependency under intubation. PS compensating tube resistance fluctuated with respiratory rate. At a respiratory rate of 24/min, the endotracheal tube did not greatly influence WOB under PSV and the regression line of WOB converged with the WOB of ASL 5000™ alone under PS 1 cmH_2_O; however, at 36/min, endotracheal tube was resistant to breathing under PSV because trigger work increased exponentially with PS ≤ 9 cmH_2_O. The regression line of WOB under PSV converged with the WOB of T-piece breathing under PS 1 cmH_2_O. Furthermore, PS compensating endotracheal tube resistance was 6 cmH_2_O. The WOB of ASL 5000™ alone approached that of respiratory distress syndrome (RDS); however, the pressure of patient effort was normal physiological range at PS 10 cmH_2_O. PS equalizing WOB under PSV with that after extubation depended on the respiratory rate and upper airway resistance. If WOB after extubation equaled that of T-piece breathing, the PS was 0 cmH_2_O regardless of the respiratory rates. If WOB after extubation approximated  to that of ASL 5000™ alone, the PS depended on the respiratory rate.

**Conclusion:**

SBT strategies should be selected per neonatal respiratory rates and upper airway resistance.

## Background

Mechanical ventilation weaning has become a common procedure in the neonatal intensive care unit. Extubation failure reportedly increases morbidity, length of hospital stay, and mortality [1]. Spontaneous breathing trials (SBTs) with pressure support (PS) are better than continuous positive airway pressure (CPAP) for adults because successful SBT rates with PS are higher than CPAP without an increase in the reintubation rate [2, 3]. There are both pros and cons to apply pressure support for spontaneous breathing trials in infant. Endotracheal tubes used in neonates are not as resistant to breathing as was originally anticipated [4–6]; therefore, spontaneous breathing trials (SBTs) with CPAP, without PS, have been recommended [7, 8]. However, SBT with PS is reportedly useful and the positive predictive value of successful extubation is 93% [9]. The PS criteria for SBTs are set at 10 cmH_2_O with 3.0- and 3.5-mm tubes or at 8 cmH_2_O with 4.0- and 4.5-mm tubes [9–12]. There are obvious discrepancies between the two theories [7, 9]. It is difficult to clinically evaluate work of breathing. This study aimed to assess the validity of these criteria for neonates.

## Methods

We conducted a lung simulation study using a high-end lung simulator to investigate the effect of reductions in PS and increase in the respiratory rate on SBTs.

### Devices

We used an IngMar ASL 5000™ artificial lung simulator (version 3.4, 3.5; IngMar Medical, Pittsburgh, PA) with a built-in cylinder with a 17.8-cm diameter. The ASL 5000™ is a popular lung stimulator, which can imitate different breathing conditions and can measure various ventilation parameters including WOB, trigger work (TW), pressure of effort (pressure of muscle [Pmus]), maximum pressure drop during trigger, and positive end-expiratory pressure (PEEP). Respiratory parameters are automatically displayed on the control panel. We regarded the ASL 5000™ as a model of the lower respiratory tract (i.e., the upper respiratory tract was not included). We set the ASL 5000™ to reflect a 3-kg neonate after cardiac surgery to simulate SBTs with compliance at 0.5 mL/cmH_2_O/kg [5] and resistance at 40 cmH_2_O/L/s. The reference values for healthy neonate compliance and resistance are 1.5–2.0 mL/cmH_2_O/kg and 20–40 cmH_2_O/L/s, respectively [13, 14]. The ASL 5000™ was set to the constant VT mode under computer control, with a tidal volume of 30 mL (10 mL/kg) and a minute volume of 720–1080 mL/min, which corresponds to a respiratory rate (f) of 24–36/min. Endotracheal tubes with an inside diameter of 3.0 and 3.5 (Mallinckrodt™; Hi-Contour Oral/Nasal Tracheal Tube Cuffed Murphy Eye, Dublin, Ireland) were clinically curved and cuffed to prevent gas leakage. A 22/19-mm adapter with a built-in duct (diameter, 9 mm) was attached because the port of the ASL 5000™ was too large to attach an endotracheal tube. A ventilator (SERVO-i Universal™, version 3.0.1; Maquet, Danvers, MA) was set at PSV: PEEP, 4 cmH_2_O; FIO_2_, 0.4; inspiration time was set at 45% of respiration; and bias flow of 0.5 L/min was continuously delivered to the respiratory circuit. Trigger sensitivity was set to 5 to detect bias flow deviation of 0.25 L/min at the expiratory channel. The ventilator was connected to the artificial lung by means of a respiratory circuit (Smooth-Bor™; Smooth-Bor Plastics, Laguna Hills, CA). No respiratory humidifier or heat/moisture exchanger was used.

### Study

The following work and pressure parameters were measured under three breathing settings: (1) ASL 5000™ alone, (2) T-piece breathing, and (3) PSV. The parameters were measured under two control settings: the respiratory rate control setting and the PS control setting. At first, the parameters of all three breathing settings were measured in the respiratory rate control setting. In the respiratory rate control setting, the respiratory rate was increased from 24 to 36/min. The parameters under PSV were measured with a fixed PS of 10 cmH_2_O and 8 cmH_2_O in the respiratory rate control setting. Then, the PS control setting was used under PSV alone. The parameters were measured under the PS control setting with a fixed respiratory rate of 24 and 36/min. Under the PS control setting, PS was decreased from 14 to 0 cmH_2_O.

### Definition of respiratory variables

WOB measurement was started at 0.5 mL of gas inhaled and ended at 0.5 mL gas exhaled. TW was defined as the WOB between the start of the WOB measurement and the point in time at which airway pressure returned to baseline (PEEP), after a downward deflection. WOB and TW were calculated using Eq. (1) (below). Patient effort was defined as the negative of muscle pressure [− Pmus], which resembles an esophageal pressure tracing. We specified that the WOB of the ASL 5000™ alone must be lower than the WOB after extubation, because the ASL 5000™ did not include the upper respiratory tract.

1 $$ \mathrm{WOB}\left(\mathrm{mJ}/\mathrm{Breath}\right)=\int \mathrm{Pmus}\left({\mathrm{cmH}}_2\mathrm{O}\right)\ \mathrm{dV} $$

WOB was measured at a stable tidal volume, and the mean and standard deviation values were determined from 10 breaths to account for instability.

Dynamic distending pressure was calculated using Eq. (2):

2 $$ \mathrm{Dynamic}\ \mathrm{distending}\ \mathrm{pressure}\left({\mathrm{cmH}}_2\mathrm{O}\right)=\mathrm{PIP}\ \left({\mathrm{cmH}}_2\mathrm{O}\right)-\mathrm{Pmus}\left({\mathrm{cmH}}_2\mathrm{O}\right) $$

Peak inspiratory pressure (PIP) is the pressure which is delivered by ventilator. Dynamic distending pressure of T-piece breathing is equivalent of Pmus of T-piece breathing, because T-piece breathing is not under pressure support.

The Reynolds number was calculated from the mean and peak flow using Eq. (3) [15].

3 $$ \operatorname{Re}=\left[2\rho \left(\mathrm{g}/{\mathrm{cm}}^3\right)\times \dot{V} \left({\mathrm{cm}}^3/\mathrm{s}\right)\right]\div \left[\pi \times r\left(\mathrm{cm}\right)\times \eta \left(\mathrm{poise}\right)\right] $$

where we used the following constants: 20 °C; dry gas, FiO_2_, 0.4, viscosity *η* (poise = 0.1 Pa s = kg/m s), 18.72 × 10^−6^, and density *ρ* (g/cm^3^), 1.231 × 10^3^ [16, 17]. *V̇* is the mean flow rate (cm^3^/s), *r* is the inner radius of the tube (cm), and *π* is the ratio of the circumference of the circle to its diameter.

### Statistical analysis

Ten successive breaths per condition were measured. We used two-way analysis of variance (ANOVA) with Tukey’s multiple-comparison test for statistical analyses. WOB and TW were analyzed by linear or non-linear regression analysis as appropriately. All statistical analyses were performed using GraphPad Prism (GraphPad Software, Inc., La Jolla, CA). A *p* value < 0.05 was considered statistically significant.

## Results

### Effect of respiratory rate on patient effort

**Fig. 1:**
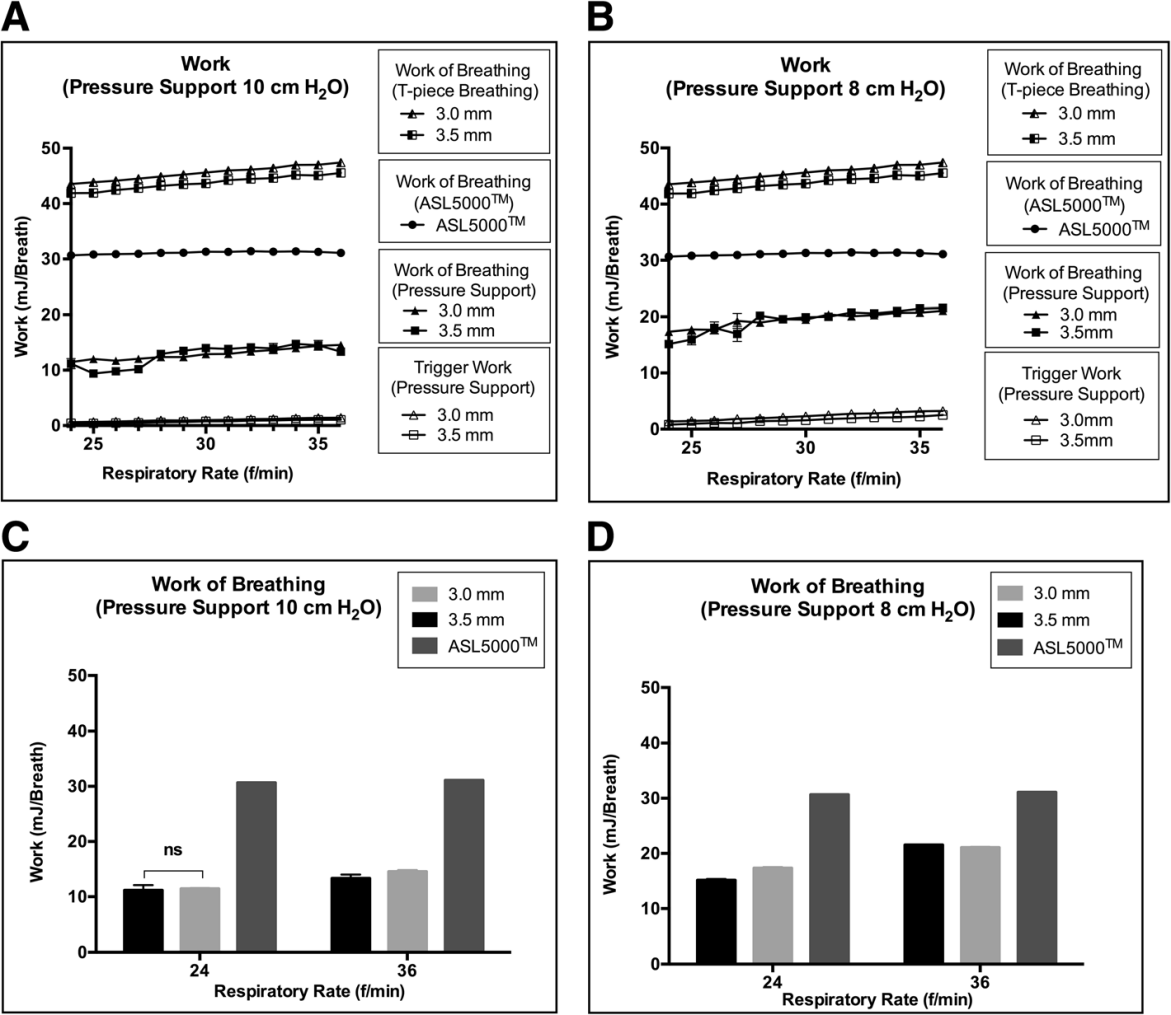
Work of breathing on an ASL 5000™ artificial lung simulator. **a**, **c** Work of breathing under pressure support of 10 cmH_2_O at a respiratory rate of 24 to 36/min. **b**, **d** Work of breathing under pressure support of 8 cmH_2_O at a respiratory rate of 24 to 36/min. **c**, **d** Comparisons between work of breathing under pressure support ventilation and ASL 5000™ alone. The error bars represent standard deviation values. If “non-significant (ns)” is not represented, then the groups are significantly different by Tukey’s multiple-comparisons test (*p* < 0.05)

**Fig. 2:**
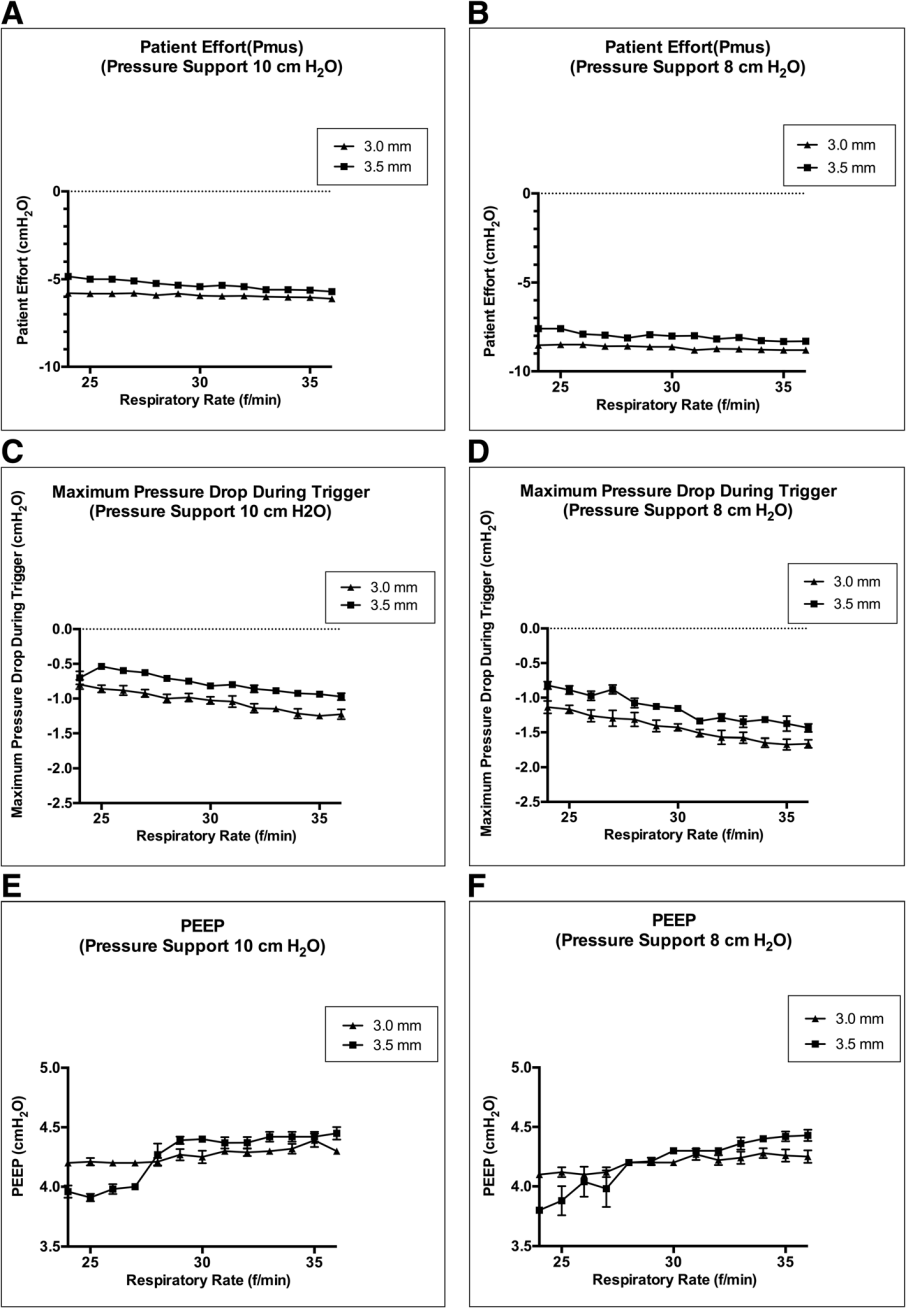
**a**, **c**, **e** Pressure parameters under pressure support of 10 cmH_2_O at a respiratory rate of 24 to 36/min. **b**, **d**, **f** Pressure parameters under pressure support of 8 cmH_2_O at a respiratory rate of 24 to 36/min. **a**, **b** Patient effort (Pmus). **c**, **d** Maximum pressure drop during trigger. **e**, **f** Positive end-expiratory pressure. The error bars represent standard deviation values

Under the respiratory rate control setting, WOB during T-piece breathing was higher than WOB of the ASL 5000™ alone and WOB of the ASL 5000™ alone was significantly higher than WOB under PS 10 cmH_2_O and PS 8 cmH_2_O in terms of physiological minute volume regardless of tube size (Fig. 1a, b). Although WOB during T-piece breathing and WOB under PS increased as the respiratory rate increased, WOB of ASL 5000™ alone did not increase as the respiratory rate increased. WOB with different tube sizes were approximately the same (Fig. 1C, D). When PS was set to 8 cmH_2_O, Pmus with 3.0–3.5-mm tubes deviated from the normal physiological range (− 3 to − 8 cmH_2_O) [18] (Fig. 2a, b), even when WOB under the PS was lower than WOB of the ASL 5000™ alone (Fig. 1a, b). Additionally, the maximum pressure drop during trigger with a 3.0-mm tube deviated from the normal range (− 0.5 to − 1.5 cmH_2_O) [19] (Fig. 2c, d). Development of auto-PEEP was dependent on the respiratory rate and tube size (Fig. 2e, f). Auto-PEEP occurred with 3.0-mm tubes at a respiratory rate of 24/min and with 3.5-mm tubes at a respiratory rate of 28/min (Fig. 2e, f). WOB increased sigmoidally as the respiratory rate increased, due to the development of auto-PEEP (Fig. 1a, b).

### Effect of pressure support on patient effort

**Fig. 3:**
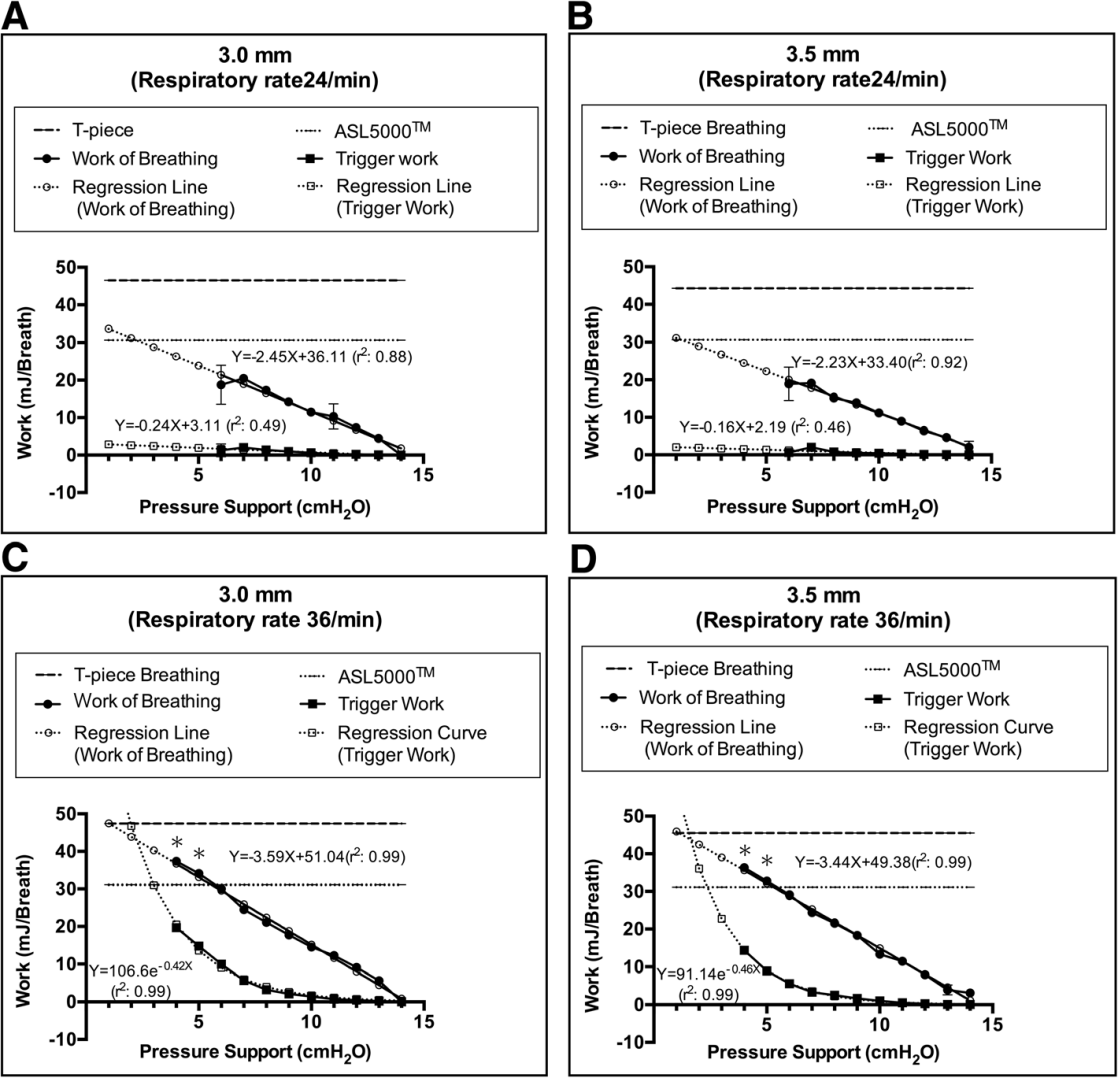
Work of breathing and trigger work under pressure support from PS 14 to 0 cmH_2_O at a respiratory rate of 24/min with 3.0-mm tubes (**a**) and with 3.5-mm tubes (**b**). Values for at a respiratory rate of 36/min with 3.0-mm tubes (**c**) and with 3.5-mm tubes (**d**). Work of breathing of T-piece breathing and ASL 5000™ alone are also described. Error bars represent standard deviation values. Asterisk denotes that the work of breathing under pressure support is significantly higher than the work of breathing of the ASL 5000™ alone (*p* < 0.05)

**Fig. 4:**
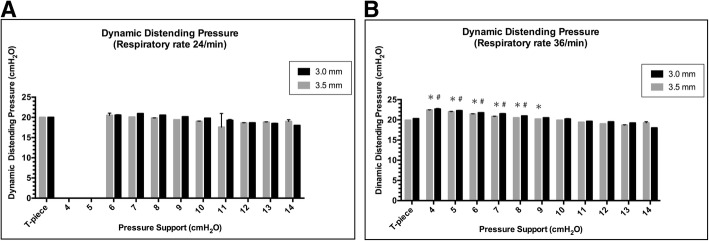
Relationship between dynamic distending pressure and pressure support at a respiratory rate of 24/min (**a**) and 36/min (**b**). Comparisons between dynamic distending pressure under pressure support and T-piece breathing tubes are presented (*p* < 0.05 by Tukey’s multiple-comparisons test). Error bars represent standard deviation values. Asterisk denotes that dynamic distending pressure of 3.5-mm tubes is significantly higher than that at T-piece breathing. Number sign denotes that dynamic distending pressure of 3.0-mm tubes is significantly higher than that at T-piece breathing

Under the PS control setting, WOB increased linearly as PS decreased from 14 cmH_2_O. Investigations were stopped at PS 5 cmH_2_O with a respiratory rate of 24/min and at PS 3 cmH_2_O with a respiratory rate of 36/min, because double-triggering and mis-triggering frequently occurred (Fig. 3). WOB increased more rapidly as PS decreased at a respiratory rate of 36/min than at a respiratory rate of 24/min (Fig. 3a–d). TW at a respiratory rate of 24/min increased linearly as the PS decreased (Fig. 3a, b). TW at a respiratory rate of 36/min increased exponentially as PS decreased from 9 to 4 cmH_2_O (Fig. 3c, d). At a respiratory rate of 36/min, WOB with 3.0–3.5-mm tubes under PS ≤ 5 cmH_2_O was higher than that of the ASL 5000™ alone (Fig. 3c–d). The regression line of WOB under PS was drawn from PS 14 to 1 cmH_2_O. The regression equation under PS was not obtained at PS 0 cmH_2_O because WOB of PS 0 cmH_2_O was not under PS ventilation, and there was a difference in the assumption (Fig. 3a–d). The regression line of WOB under PSV at a respiratory rate of 24/min converged to WOB of ASL 5000™ alone as PS decreased (Fig. 3a, b). The regression line of WOB under PSV intersected with WOB of ASL 5000™ alone at PS 2.5 cmH_2_O with the 3.0-mm tube and PS 1.2 cmH_2_O with the 3.5-mm tube. The regression line of WOB and TW at a respiratory rate of 36/min converged to WOB of T-piece breathing as PS decreased (Fig. 3c, d). The regression line of WOB under PSV at a respiratory rate of 36/min intersected with WOB of T-piece breathing at PS 1.1 cmH_2_O with the 3.0–3.5-mm tube. Dynamic distending pressure at a respiratory rate of 24/min showed a result similar to that obtained with T-piece breathing regardless of PS (Fig. 4a). Dynamic distending pressure at a respiratory rate of 36/min using PS ≤ 9 cmH_2_O with 3.5-mm tubes and PS ≤ 8 cmH_2_O with 3.0-mm tubes was higher than that with T-piece breathing (Fig. 4b).

### Effect of flow rate on patient effort

**Fig. 5:**
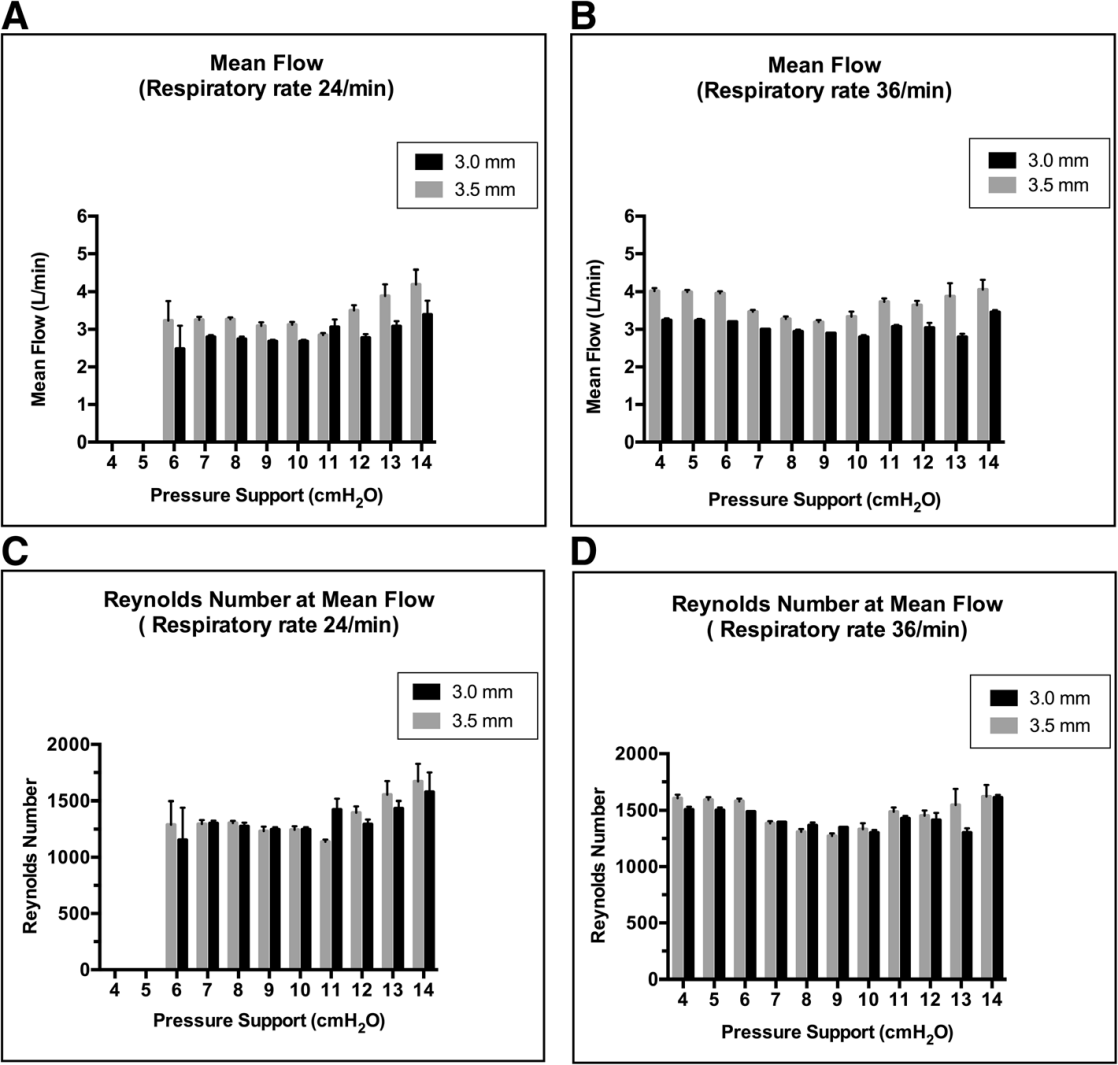
Relationship between mean flow and pressure support at a respiratory rate of 24/min (**a**) and 36/min (**b**). Relationship of Reynolds number and pressure support at a respiratory rate of 24/min (**c**) and 36/min (**d**) (*p* < 0.05 by Tukey’s multiple-comparisons test). Error bars represent standard deviation values

Maximum mean flow was 4.1 ± 0.2 L/min with 3.5-mm tubes and 3.5 ± 0 L/min with 3.0-mm tubes under PS 14 cmH_2_O at a respiratory rate of 36/min (Fig. 5a, b). The Reynolds number at mean flow was < 1760: 1619 ± 98 with 3.5-mm tubes; and 1610 ± 22 with 3.0-mm tubes at PS 14 cmH_2_O (Fig. 5c, d). Reynolds number values at peak flow were > 2000: 3737 ± 25 with 3.5-mm tubes and 3308 ± 32 with 3.0-mm tubes at PS 14 cmH_2_O. During T-piece breathing, mean flow was 3.3 ± 0 L/min with 3.5-mm tubes, and 3.1 ± 0 L/min with 3.0-mm tubes, at a respiratory rate of 36/min. The Reynolds number at mean flow was < 1760: 1316 ± 0 with 3.5-mm tubes and 1447.3 ± 14 with 3.0-mm tubes during T-piece breathing. All Reynolds number values at peak flow were > 2000: 2672.6 ± 0 with 3.5-mm tubes and 2750 ± 2.9 with 3.0-mm tubes during T-piece breathing. Mean flow was 3.6 ± 0.1 L/min with the ASL 5000™ alone. With the ASL 5000™ alone, the Reynolds number values were 227.8 ± 4.4 at mean flow and 505.8 ± 2.9 at peak flow, at a respiratory rate of 36/min.

## Discussion

**Fig. 6:**
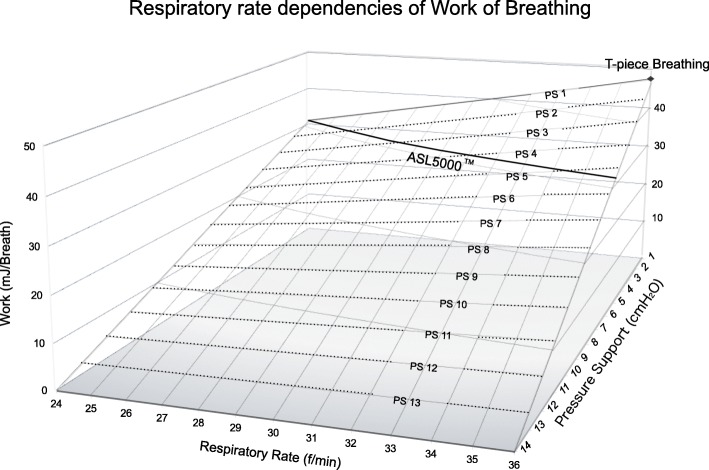
Work of breathing (WOB) under pressure support (PS) is denoted by the dotted line, and ASL 5000™ alone is denoted by a solid line. Black diamond denotes WOB of T-piece breathing

This study reports a respiratory rate dependency of WOB under intubation. At a respiratory rate of 24/min, the endotracheal tubes may not greatly affect WOB under PSV because the regression line of WOB under PSV converged with that of ASL 5000™ under PS 1 cmH_2_O. At a respiratory rate of 36/min, endotracheal tubes were resistant to breathing because TW increased exponentially under PS ≤ 9 cmH_2_O. The regression line of WOB under PSV converged with that of T-piece breathing under PS 1 cmH_2_O. According to the two regression lines, if the regression line of WOB under PS 1 cmH_2_O connects WOB of ASL 5000™ alone at a respiratory rate of 24/min with WOB of T-piece breathing at a respiratory rate of 36/min, the three-dimensional surface of the WOB under PSV with 3.5-mm tubes is approximated by Eq. (4) (Fig. 6). f_B_ is the respiratory rate per min.

4 $$ \mathrm{WOB}\left(\mathrm{mJ}/\mathrm{Breath}\right)=-\left(1.24{\mathrm{f}}_B\left(\mathrm{f}/\min \right)+0.89\right)\left(\mathrm{PS}\left({\mathrm{cmH}}_2\mathrm{O}\right)-14\right)/13 $$

Assuming that the WOB of ASL 5000™ alone is fixed at 30.7 mJ/Breath, which is the WOB of ASL 5000™ alone at a respiratory rate of 24/min, the PS compensating 3.5-mm tube resistance is approximated by the following equation as a function of respiratory rate (5).

5 $$ \mathrm{PS}\left({\mathrm{cmH}}_2\mathrm{O}\right)=14-399.1/\left(1.24{\mathrm{f}}_B\left(\mathrm{f}/\min \right)+0.89\right) $$

Therefore, the PS compensating tube resistance fluctuates with the respiratory rate.

Upper airway resistance is similarly dynamic, depending on nasal breathing, mouth breathing, or respiratory support [20]. Nasal airway resistance accounts for approximately two thirds of total upper airway resistance, and the resistance is comparable to that of the 3.0–3.5-mm tube [6]. However, the glottis and larynx contribute to less than 10% of total upper airway resistance [21]. PS equalizing WOB under PSV with that after extubation depended on the respiratory rate and upper airway resistance. If WOB after extubation equaled that of T-piece breathing, the PS was 0 cmH_2_O regardless of the respiratory rates. If WOB after extubation approximated to that of ASL 5000™ alone, the PS depended on the respiratory rate. Minimum PS is adequate for neonates in a better condition, requiring a lower respiratory rate; however, PS compensating tube resistance may be necessary for neonates in marginal respiratory conditions, requiring higher respiratory rates. SBTs with PS 10 cmH_2_O are so potent that patient effort is decreased to normal physiological range under respiratory distress syndrome (RDS)-like conditions regardless of tube size [5, 22, 23]. Extubation is not recommended for neonates intolerant to SBTs even at PS 10 cmH_2_O. At a respiratory rate of 36/min with 3.0–3.5-mm tubes, the pressure of patient effort exceeded the physiological range under PS 8 cmH_2_O even when WOB under PSV was lower than that after extubation. Furthermore, it is necessary to evaluate patient effort to assess SBTs. Tachypnea and flow starvation may impose non-physiological stress on the lungs [24].

Furthermore, the Reynolds number at mean flow was < 1760 and was > 2000 at peak flow under intubation. Therefore, gas flow became turbulent at peak flow and then decelerated markedly to a laminar flow, regardless of tube size, because the lower critical Reynolds number is approximately 1760, below which turbulent structures cannot be sustained by any induced disturbance [25]. The pressure gradient of turbulent flow produces fluid flow less efficiently than that of laminar flow [26–28], which may have affected WOB and pressure parameters. The upper critical value of the Reynolds number for transition from a laminar to a turbulent flow cannot be generalized even when at 2000 in clinical practice. Hof et al. [28] reported that “Most pipe flows are turbulent in practice even at modest flow rates”. The inlet diameter of ASL 5000™ was sufficiently large, such that inspiratory flow constantly remained laminar with an increase in respiratory rate because the Reynolds number was constantly < 1760. These results potentially explain why WOB of ASL 5000™ alone did not increase with an increase in respiratory rate in addition to the difference in inlet diameter and absence of tube length resistance.

### Limitations

The ASL 5000™ is an artificial lung model that excludes the upper respiratory tract. In our study, we could not determine the pressure at which WOB would be equivalent to WOB after extubation. The tidal volume of the ASL 5000™ can be set in increments of 10 mL. We considered the physiological tidal volume to be 5–8 mL/kg. A tidal volume of 20 mL cannot cover 8 mL/kg (24 mL for a 3-kg infant); therefore, we chose to use a tidal volume of 30 mL. The upper limit of respiratory rate was determined by dividing the physiological minute volume of 1080 mL/min by the tidal volume of 30 mL. We added PEEP at the minimum required value of 4 cmH_2_O for neonates, which was lower than that generally used during SBTs. However, there was no atelectasis or tidal recruitment in the ASL 5000™; therefore, the PEEP value of 4 cmH_2_O seemed to have minimal impact on this study. Gas is heated and humidified in a clinical setting, but humidified gas could not be used for the ASL 5000™. Water vapor has a lower density and viscosity than oxygen or nitrogen; kinetic viscosity (*η*/*ρ* [m^2^/s]) increased from 15.1 × 10^−6^ to 16.6 × 10^−6^ with heating and humidification of dry air at 20 °C to relative humidity of 100% at 37 °C, and the Reynolds number decreased by approximately 9%. Therefore, heating and humidification may not greatly affect fluid characteristics [17, 26, 29].

## Conclusions

WOB displayed respiratory rate dependency under intubation. We should judge which strategy is appropriate for neonates in various respiratory conditions.

## Abbreviations

ANOVA: Analysis of variance
CPAP: Continuous positive airway pressure
Pao: Airway opening pressure
PEEP: Positive end-expiratory pressure
PIP: Peak inspiratory pressure
Pmus: Pressure of muscle
Ppl: Pleural space
PS: Pressure support
PSV: Pressure support ventilation
SBT: Spontaneous breathing trial
TW: Trigger work
WOB: Work of breathing
